# A Microbiomic Analysis in African Americans with Colonic Lesions Reveals *Streptococcus* sp.VT162 as a Marker of Neoplastic Transformation

**DOI:** 10.3390/genes8110314

**Published:** 2017-11-09

**Authors:** Hassan Brim, Shibu Yooseph, Edward Lee, Zaki A. Sherif, Muneer Abbas, Adeyinka O. Laiyemo, Sudhir Varma, Manolito Torralba, Scot E. Dowd, Karen E. Nelson, Wimal Pathmasiri, Susan Sumner, Willem de Vos, Qiaoyi Liang, Jun Yu, Erwin Zoetendal, Hassan Ashktorab

**Affiliations:** 1 Department of Pathology, Department of Medicine, Department of Biochemistry & Molecular Biology, Department of Microbiology and Cancer Center, College of Medicine, Howard University, 2041 Georgia Avenue, Washington, DC 20060, USA; ellee@howard.edu (E.L.); zaki.sherif@howard.edu (Z.A.S.); m_abbas@howard.edu (M.A.); adeyinka.laiyemo@howard.edu (A.O.L.); hashktorab@howard.edu (H.A.); 2 J. Craig Venter Institute, La Jolla, CA 92037, USA; syooseph@jcvi.org (S.Y.); mtorralba@jcvi.org (M.T.); 3 HiThru Analytics, Laurel, MD 20877, USA; sudhir.varma@hithru.com; 4 MRDNA/Molecular Research LP, Shallowater, TX 79363, USA; sdowd@mrdnalab.com; 5 J. Craig Venter Institute, Rockville, MD 20850, USA; knelson@jcvi.org; 6 Systems and Translational Sciences, RTI International, NC 27709, USA; wpathmasiri@rti.org (W.P.); ssumner@rti.org (S.S.); 7 Laboratory of Microbiology, Department of Agrotechnology and Food Sciences, Wageningen University, 6708 PB Wageningen, The Netherlands; willem.devos@wur.nl (W.d.V.); erwin.zoetendal@wur.nl (E.Z.); 8 Institute of Digestive Disease and Department of Medicine and Therapeutics, State Key Laboratory of Digestive Disease, Li Ka Shing Institute of Health Sciences, CUHK Shenzhen Research Institute, The Chinese University of Hong Kong, Hong Kong, China; JessieQY@cuhk.edu.hk (Q.L.); junyu@cuhk.edu.hk (J.Y.)

**Keywords:** colorectal cancer, microbiota, African Americans, metabolomics, metagenomic linkage groups

## Abstract

Increasing evidence suggests a role of the gut microbiota in colorectal carcinogenesis (CRC). To detect bacterial markers of colorectal cancer in African Americans a metabolomic analysis was performed on fecal water extracts. DNA from stool samples of adenoma and healthy subjects and from colon cancer and matched normal tissues was analyzed to determine the microbiota composition (using 16S rDNA) and genomic content (metagenomics). Metagenomic functions with discriminative power between healthy and neoplastic specimens were established. Quantitative Polymerase Chain Reaction (q-PCR) using primers and probes specific to *Streptococcus* sp. VT_162 were used to validate this bacterium association with neoplastic transformation in stool samples from two independent cohorts of African Americans and Chinese patients with colorectal lesions. The metabolomic analysis of adenomas revealed low amino acids content. The microbiota in both cancer vs. normal tissues and adenoma vs. normal stool samples were different at the 16S rRNA gene level. Cross-mapping of metagenomic data led to 9 markers with significant discriminative power between normal and diseased specimens. These markers identified with *Streptococcus* sp. VT_162. Q-PCR data showed a statistically significant presence of this bacterium in advanced adenoma and cancer samples in an independent cohort of CRC patients. We defined metagenomic functions from *Streptococcus* sp. VT_162 with discriminative power among cancers vs. matched normal and adenomas vs. healthy subjects’ stools. *Streptococcus* sp. VT_162 specific 16S rDNA was validated in an independent cohort. These findings might facilitate non-invasive screening for colorectal cancer.

## 1. Introduction

Colorectal cancer (CRC) is the third most prevalent cancer worldwide [1,2,3]. It is more prevalent in the West than in other part of the World. In the US, there are discrepancies within different ethnic/racial groups [4,5]. African Americans (AAs) have a high incidence of, and mortality from this disease [6,7]. Several factors have been proposed and investigated, including genetics, epigenetics, diet, socio-economic status and access to healthcare [8,9,10,11,12,13,14,15].

Several publications suggest an essential, mutualistic relationship between the host and their colonic microbiota [16,17,18]. A single commensal, *Bacteroides thetaiotamicron,* was shown to induce colonic mucosal gene expression, angiogenesis and immune responses in mouse models of colon cancer, revealing a broader extent of microbe-mucosal communication and cross-regulation than previously recognized [19]. Similar findings were also obtained in colorectal cancer mouse models with enterotoxigenic *Bacteroides fragilis* [20,21]. The human colon harbors the greatest number and diversity of organisms, primarily bacteria, than any other organ in the human body [22,23]. Molecular analysis of the colonic luminal and mucosal microbiota indicates that individuals harbor unique microbiotas that are fairly stable along the colonic axis. However, the mucosal microbiota is either distinct or contains only a subset of the bacterial phylotypes identified in the luminal fecal samples [24,25].

The primary local environment to which the colonic mucosa is exposed is created by the microbiota of the colon and their metabolic products that include beneficial non-carcinogenic components such as short-chain fatty acids as well as harmful compounds including toxins and other proliferation-promoting metabolites [26,27,28]. Two divisions of bacteria (*Bacteroidetes* and *Firmicutes*) are considered dominant in the cultured colonic microbiota. *Actinobacteria* were also reported as prevalent in the intestinal tract but their presence has been underestimated in Polymerase Chain Reaction (PCR)-based approaches [29].

Several recent studies, using next generation sequencing technologies, have set the framework for metagenomic studies in general and for the gut microbiota in particular [24,25,30,31,32,33,34,35,36]. Huge databases for 16S rRNA genes as well as for gut microbiota functions have been established as a resource for other studies in the field [37,38,39]. We capitalized on such data to run an analysis of stool samples from AAs with colon polyps that showed subtle differences in the microbiota composition at the Operational Taxonomic Units (OTUs) level when compared to healthy individuals [40]. While this published study further reinforced the presence of oncogenic-associated microbiota’s changes, it lacked the potential to define bacterial markers with diagnostic potential that might directly affect the colon mucosa and that might serve for screening purposes.

In this study, we performed a microbiomic study in AAs with colorectal lesions. Bacterial markers of potential diagnostic value were defined and validated in an independent cohort.

## 2. Materials and Methods

### 2.1. Ethics Statement

The present study was approved by the Howard University Institutional Review Board (IRB-06-MED-39). Written consent forms were obtained from all participants.

### 2.2. Sample Collection and Preparation

Cancer and matched-normal tissues from 10 AAs who underwent surgical resection at Howard University Hospital were collected through our Pathology Department after pathological evaluation of the surgical specimens by a gastrointestinal (GI) pathologist (E.L.). The samples were snap frozen and stored at −80 °C until DNA extraction. The samples were chosen to equally represent right and left side colon sections. Twenty stool samples were also collected from 10 colon adenoma patients and 10 healthy subjects (colorectal lesions-free). The patients were given stool collection containers on the day of the colonoscopy and were requested to collect stool samples aseptically. The stool samples were collected two months after the colonoscopy, delivered on the same day of collection to our laboratory and stored immediately as previously described [40].

### 2.3. Metabolomics Analysis

NMR (Nuclear Magnetic Resonance) metabolomic analysis [41,42] was performed on aqueous fecal extracts obtained from 10 healthy individuals and 10 colon adenoma patients. Briefly, fecal samples (20 mg) were mixed with 1000 μL of D_2_O (Deuterium Oxide/Heavy water), mixed thoroughly by vortexing, and then centrifuged at 16,000 rcf for 20 min. The supernatants were removed and filtered through 0.22 μL centrifuge filters at 16,000 rcf for 20 min. A 540 μL aliquot of the filtrate was mixed with 60 μL of Chenomix Internal Standard solution (containing 4,4-dimethyl-4-silapentane-1-sulfonic acid (DSS, chemical shift reference), Imidazole (pH reference), and NaN_3_ (to inhibit bacterial growth) in D_2_O) and vortexed for 30 s. Aliquots from the supernatant (550 μL) were then transferred into 5 mm NMR tubes. NMR data were acquired using a Bruker Avance III 950 MHz NMR spectrometer (Bruker-Biospin, Rheinstetten, Germany) located at the David H. Murdock Research Institute in Kannapolis, NC. Standard NMR spectra were acquired at 25 °C with a standard one-dimensional pulse sequence of a NOESY scheme with water suppression using a relaxation delay of 2 s and a mixing time of 100 ms. A total of 256 transients were collected into 32,768 data points for each spectrum with a spectral width of 16 ppm. Free induction decays were zero filled and Fourier transformed using exponential multiplication with a 0.5 Hz line-broadening factor. ^1^H-NMR spectra were manually phased and baseline-corrected by using Topspin 3.0 (Bruker Biospin, Rheinstetten, Germany). The ^1^H-NMR spectra were referenced to the DSS at δ 0.0. NMR data were processed for metabolomics analysis by automated integration using a 0.04 ppm bin width over the range of δ 0.5–10.0 ppm using Chenomx NMR Suite 7.5 Professional (Chenomx, Edmonton, AB, Canada) after excluding the regions of water suppression, and imidazole. The bin integrals were normalized to the total integral of each spectrum, mean centered, and Pareto scaled prior to multivariate data analyses. Multivariate analysis was performed using the SIMCA-P+ (version 12, Umetrics AB, Umeå, Sweden) software package. Initial Principal Component Analysis (PCA) and subsequent Projection to Latent Structure Discriminant Analysis (PLS-DA) were performed on binned data. Loadings and Variable Influence on Projection (VIP) plots in the PLS-DA were examined to determine bins that best define group separation. The NMR that had a VIP ≥ 1.0 with a jack-knife confidence interval that did not include 0 were determined to be important for differentiating the study groups. The VIP statistic summarizes the importance of the bin in differentiating the phenotypic groups, while the loadings plots provide additional information on the directionality. All models used a 7-fold cross-validation to assess the predictive variation of the model (Q2) [43,44]. NMR bins that were important for the separation of adenoma patients from control healthy subjects were matched to metabolites by using the built-in metabolite library in the Chenomx NMR Suite (Chenomx, Edmonton, AB, Canada).

### 2.4. DNA Extraction

DNA from stool samples was extracted using a Qiagen Stool DNA extraction kit and processed as previously described [40]. The extracted stool DNA was investigated for 16S rRNA gene-based bacterial diversity using a Human Intestinal Tract microarray (HITChip) as previously described [40]. The tissue samples (cancers and matched normal) were first lysed for homogenization. Then, 3 cycles of freeze-thawing were performed followed by bead-beating to achieve bacterial cell disruption. DNA from the resulting lysates was extracted using the Qiagen AllPrepDNA/RNA kit (Qiagen, Inc., Germantown, MD, USA).

### 2.5. 16S rRNA Gene Profiling

The 16S rRNA gene analysis was performed for stool samples and colon tissue samples at different times using two different comparable technologies, namely HITChip microarray and Next Generation Sequencing (NGS), respectively. We and others have previously analyzed samples simultaneously, using both technologies, and have reported that the outcomes were similar and comparable [40,43].

### 2.6. HITChip Analysis

DNA extracts from the 20 stool samples (adenoma and healthy subjects) were processed on a HITChip that contains 6000 oligonucleotide probes representing over 1000 identified intestinal bacterial species [44]. Briefly, 20 ng of DNA from each sample was used to amplify the nearly full 16S rRNA genes. PCR products were labeled with Cy3 and Cy5 and subsequently fragmented. Hybridizations were performed in duplicate, and data were extracted from microarray-scanned images using Agilent Feature Extraction software version 10.7.3.1 (http://www.agilent.com). Array normalization was performed using a set of R-based scripts (http://r-project.org) in combination with a custom designed relational database which runs under the MySQL database management system (http://www.mysql.com). Hierarchical clustering of probe profiles was carried out by calculating a distance matrix between the samples based on the squared difference between each pair of profiles (Euclidean distance). The distance matrix was used in the hclust implementation in R of a hierarchical clustering algorithm. The agglomeration method used in this algorithm was Ward’s minimum variance method. The bacterial composition was compared at the phylum level (divided into class level for the Firmicutes) and at the genus level using the Wilcoxon signed-rank test that was corrected for multiple comparisons (*p* value), in which *p* < 0.05 was considered significantly different [40].

### 2.7. 454 Pyrosequencing 16S rRNA Gene Profiling from Tissue Samples

DNA extracts from the 20 tissue samples (10 cancers and their normal matched) were amplified using primers that targeted the V1–V3 region of the 16S rRNA genes [45]. These primers included the A and B adaptor sequences for 454 pyrosequencing as well as a unique 12 bp barcode incorporated onto the reverse primer such that each sample received its own unique barcode. Using approximately 100 ng of extracted DNA, the amplicons were generated with Platinum *Taq* polymerase (Invitrogen, CA, USA) using the following cycling conditions: 95 °C for 5 min for an initial denaturing step followed by 35 cycles of: 95 °C for 30 s, 55 °C for 30 s, 72 °C for 30 s, followed by a final extension step of 72 °C for 7 min, and then stored at 4 °C. Once the PCR for each sample was completed, the amplicons were purified using the QIAquick PCR purification kit (Qiagen Valencia, CA, USA), quantified, normalized, and then pooled in preparation for emulsion PCR and 454 sequencing using Titanium chemistry (Roche, Basel, Switzerland) according to the manufacturer’s protocol. The generated sequencing data were first deconvoluted using the sample barcodes to identify sequences from each of the samples. Barcode, primer, and adaptor sequences were trimmed as part of this step. The resulting deconvoluted sequence data were processed using the mothur program [46], which was used to assign taxonomy and also to generate clusters of Operational Taxonomic Units (OTUs) at 97% sequence identity [47].

### 2.8. Metagenomic Analysis

Data generation: Prepared DNA from the 40 analyzed samples (20 stool samples: 10 adenomas/10 healthy subjects and 20 tissue samples: 10 cancers/10 matched normal) was processed in preparation for library construction for MiSeq Illumina sequencing. The library for each sample was prepared using the Nextera DNA Sample Preparation Kit (Illumina, San Diego, CA, USA) following the manufacturer’s instructions. The initial concentration of genomic DNA (gDNA) was measured using the Qubit^®^ dsDNA HS Assay Kit (Life Technologies, Waltham, MA, USA). All 40 samples were diluted accordingly to achieve the recommended DNA input of 50 ng at a concentration of 2.5 ng/μL. Subsequently, the samples underwent the simultaneous fragmentation and addition of adapter sequences. These adapters were added during a limited-cycle (5 cycles) PCR in which unique indices were added to the sample. Following the library preparation, the final concentration of each library was measured using the Qubit^®^ dsDNA HS Assay Kit (Life Technologies), and the average library size (around 1000 bp) was determined using Experion (Bio-Rad). The library (15 pM) was sequenced by using the 300 Cycles v2 Reagent Kit in MiSeq (Illumina). It is worth noting that sequencing was performed at 3 million reads for the stool sample DNA and 15 million reads for the tissue sample DNA to compensate for the high host’s DNA presence in tissue-extracted DNA.

Paired sequencing reads were joined and entered into a metagenomic pipeline (MG-RAST) for metagenome analysis. Supplementary Table S2a,b shows the total number of reads and the number of reads that did not map to the human genome and are thus considered of microbial origin. The data were compared to M5nr [48], a non-redundant database containing protein sequences and annotations from multiple sources, using a maximum e-value of 0.00001, a minimum identity of 60%, and a minimum alignment length of 15 amino acids for protein databases. The generated data were filtered, and all reads mapping to the human genome were removed prior to data analysis.

Assembly of de-novo metagenome genes: To create a genomic scaffolding that can be used to assign taxonomic and functional annotation to reads, we assembled putative microbial genes using the reads that did not map to the human genome. These reads were assembled into new microbial contigs using SOAPdenovo2.1. Reads from each sample were assembled separately, since we had no evidence that the metagenome distribution is the same in each subject. Then, we used MetaGeneMark [49], a modification of GeneMark 2 for metagenomic genes, to predict which contigs contain protein-coding genes. The set of predicted genes from each sample was combined to form the full set of 638,927 putative new metagenomic genes for our patient population. We combined these genes with the catalog of 4,267,985 genes from a publicly available microbiome gene catalogue [50]. We mapped the de-replicated reads to the genes in the gene catalog using the SOAP2.3 short read mapper. Reads that mapped to multiple genes were not filtered out.

Taxonomic and functional annotation: We annotated the assembled genes by sequence alignment with the M5nr database using translated protein BLAST (*blastx* with an e-value cutoff of 0.1). Note that this process cannot be used with the raw reads since the short-read aligners used with sequenced reads are not flexible with respect to number of gaps and mismatches. That motivated our approach of assembling the reads into genes and then annotating the genes. The annotated genes from both sets of data were cross-mapped to identify common sequences. These overlapping sequences were analyzed for their potential discriminative power between control samples (normal tissues and stools of healthy individuals) and specimens from diseased patients (cancer tissues and stools from adenoma patients). This analysis led to metagenomic markers from a specific *Streptococcus* strain.

### 2.9. Streptococcus sp. VT_162 Q-PCR

To further confirm *Streptococcus* sp. VT_162 association with colorectal neoplastic transformation, we used two independent validation sets from the US and Hong Kong. These sets correspond to DNA stool samples from 90 normal, 24 polyps, and 74 adenomas in the US validation set and 85 normal, 69 adenomas, 78 advanced adenomas and 88 cancers in the Hong Kong validation set. The following *Streptococcus* sp. VT_162 16S rDNA primers and probe were used: F-Primer: AGCGGCTCTCTGGCTTGTAA, R-Primer: CCCCGGAAAGGGTCTAACAC, Probe: Fam-CCCTGGTAGTCCACGCCGTAAACGA-TAMRA. Duplex Q-PCR reactions detecting *Streptococcus* sp. VT_162 and an internal control were performed as described in our previous study [51]. The primer-probe set was specific to *Streptococcus*_162 according to blast searching in the database, at the time of the primer-probe design in December 2016. Furthermore, PCR products were subjected to Sanger sequencing to confirm PCR amplification specificity. The Mann-Whitney test was used to establish the statistical significance of the q-PCR data between normal and colon lesion samples.

## 3. Results

### 3.1. Metabolomic Analysis of Adenoma Patients and Healthy Subjects’ Stool Samples

**Figure 1:**
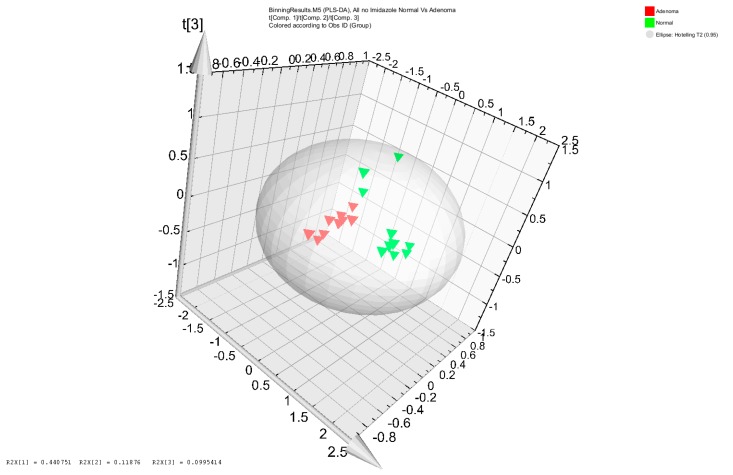
Score plot of Partial Least Squares Discriminant Analysis (PLS-DA) distinguishing adenoma patients (red triangles) from healthy subjects (black triangles).

The NMR metabolomic analysis differentiated the metabolomic profiles from stool samples of 10 colon adenoma patients and from 10 healthy subjects with no colorectal lesions (Figure 1). Upon clustering the samples’ profiles, all 10 adenoma profiles grouped together. The same was true for 7 out of 10 healthy subjects’ stool samples metabolomes. The 3 remaining healthy metabolomic profiles were scattered (Figure 1). The NMR bins that were deemed to be important for the differentiation of adenoma patients from control healthy subjects were matched to metabolites. Fatty acids (acetate, butyrate, caprate, isovalerate, and propionate), amino acids (alanine, glutamate, glycine, isoleucine, leucine, lysine, phenylalanine, threonine, and valine), lipids (including O-Phosphocholine), bile acids (cholate), and energy metabolites (glucose, glycerol, and lactate) were among the metabolites that differentiated the adenoma patients from healthy controls. More specifically, valerate, isovalerate, acetate, propionate and butyrate were more represented in the adenoma samples, while glycerol, glucose, fatty acids and several amino acids (valine, glycine, threonine and lysine) were highly represented in the healthy subjects’ stool extracts (Supplementary Table S1).

### 3.2. 16S rRNA Gene Analysis

**Figure 2:**
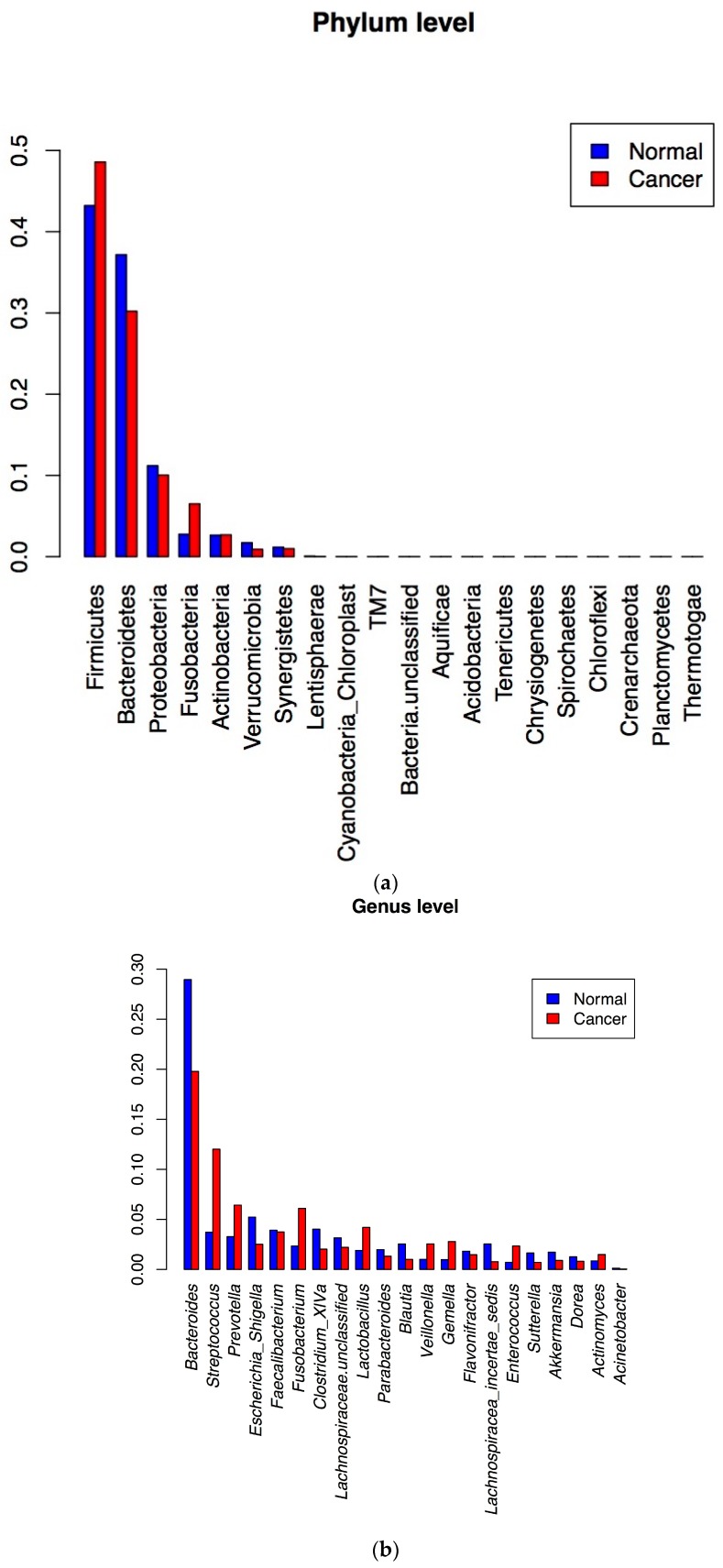

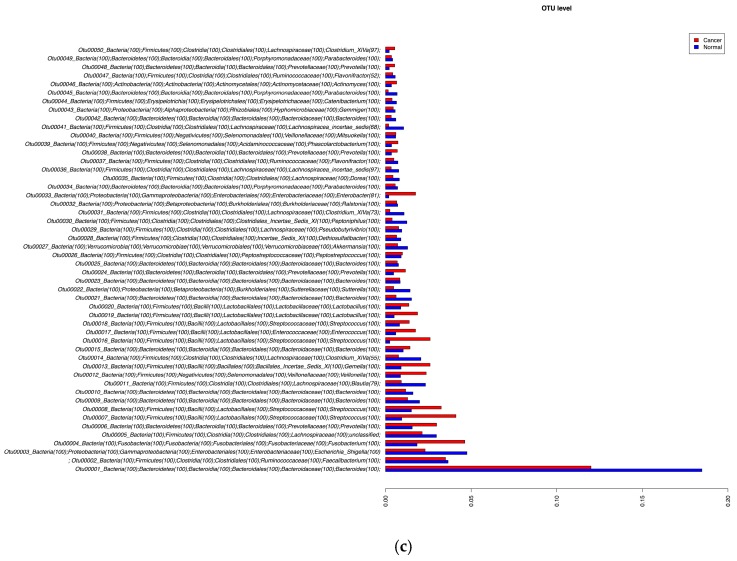
Microbiota analysis of the analyzed tissue samples at the Phylum (**a**), Genus (**b**) and Top 50 Opeartional Taxonomic Units level (**c**).

For the cancer and matched normal tissue samples, there was an average of 25,000 generated 16S RNA gene sequences per sample with a window of 15,000 to 35,000 reads after chimera filtering. The analysis of these data revealed that at the phylum level, *Firmicutes* and *Fusobacteria* were more prevalent in cancer tissues while *Bacteroides*, *Proteobacteria*, and *Verrucomicrobia* were prevalent in the matched normal tissues (Figure 2a). This differential bacterial prevalence was observed at the genus level, with the significant differences of the top 20 genera depicted in Figure 2b. The genera *Streptococcus*, *Prevotella*, *Fusobacteria*, *Lactobacillus*, *Veillonella*, *Gemella*, *Enterococcus* and *Actinomyces* were more represented in the cancer samples (Figure 2b). Figure 2c further details these differences at the OTU levels with the most striking differences noted for *Streptococcus*, *Prevotella*, and *Fusobacteria* species.

**Figure 3:**
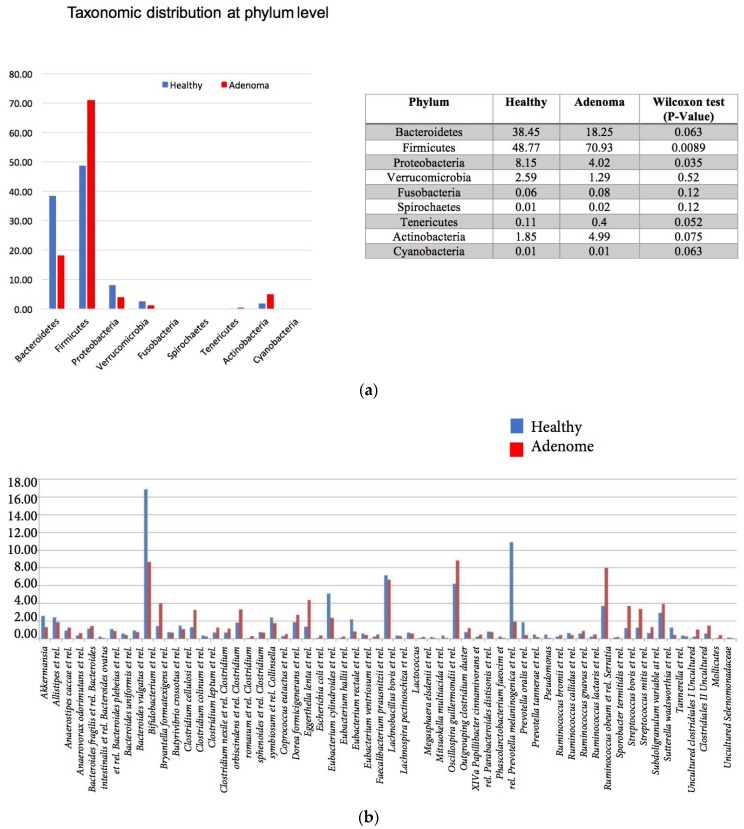
Microbiota composition of the analyzed stool samples at the Phylum (**a**) and Genus level (**b**).

As for the adenoma patients and healthy subjects’ stool samples, 16S rRNA gene analysis revealed a prevalence of *Firmicutes* and *Actinobacteria* in adenoma samples while *Bacteroides*, *Proteobacteria* and *Verucomicrobia* were more prevalent in the healthy subjects’ stool samples at the phylum level (Figure 3a). These differences were statistically significant for *Firmicutes* and *Proteobacteria*. *Fusobacteria* were almost undetectable in both sets of stool samples. The genus level distribution further detailed the phylum composition (Figure 3b). While *Prevotella* and *Bacteroides* were more dominant in the healthy subjects’ stool samples, *Bifidobacterium*, *Ruminococcus obeum*, *Streptococcus bovis* et. rel. and *Streptococcus mitis* et. rel. were prevalent in the adenoma patients’ stool samples’ bacterial communities (Figure 3b).

### 3.3. Metagenomic Data Analysis

Details of the sequenced, analyzed and annotated metagenomic data are summarized for each sample in Supplementary Table S2. While read numbers were higher in tissue samples, most were of human origin as these were generated from tissue sample DNA extracts in contrast to the stool samples’ reads, of which the majority were assembled into bacterial genes. Also, a sizable portion of the reads did not match sequences in available bacterial genome databases, further reducing the number of reads. This might also be due to the fact that the samples originate from African Americans, who might have different microbiotas than those already catalogued from other populations.

**Figure 4:**
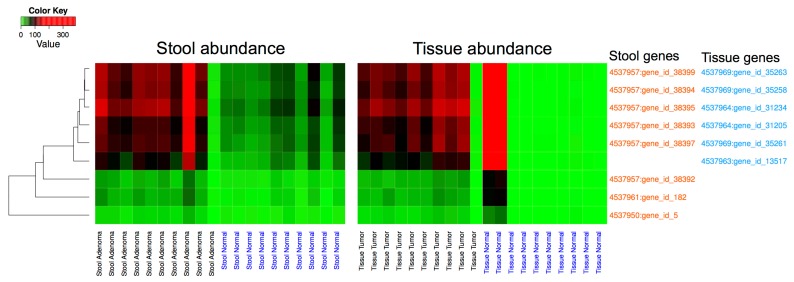
Determination of genes from stool and tissue metagenomic data with discriminative power of lesions from normal samples: (A) Stool sequencing reads mapped to 30 genes from tissue data, 6 of which discriminated between adenoma vs. Healthy subjects (A1) and cancer vs. matched normal samples (A2). Tissue sequencing data mapped to 4233 genes from stool gene data, 8 of which discriminated between adenoma vs. healthy subjects (A1) and cancer vs. matched normal samples (A2).

The annotated metagenomic data from stool and tissue samples were cross-mapped to establish common overlapping sequences between the two sets of samples. The stool reads mapped to 30 tissues’ microbiota genes, 6 of which had large enough variation (variance > 100) in the abundance of mapped stool reads to be further investigated. Similarly, the reads from the tissue samples were mapped to a total of 4233 genes from the stool samples’ dataset, of which 8 had large enough variation in the abundance of mapped tissue reads (variance > 100). These 14 genes, 6 from the tissue dataset and 8 from the stool dataset, showed a remarkable ability to discriminate between disease and normal samples in both datasets (Figure 4). The discrimination is statistically significant in the case of the stool dataset (adjusted *p* = 0.005) and visually striking, but non-significant in the tissue dataset. The 14 genes were further analyzed and were found to have 5 duplicates, resulting in a total of 9 unique sequences. In a first analysis, these sequences mapped to *Streptococcus*, *Acinetobacter*, and *Sphingomonas*. Further analysis against available bacterial genomes revealed that these 9 sequences map specifically to *Streptococcus* sp. VT_162, *Acinetobacter baumanii* AC12 and *Sphingomonas* sp. PM2-P1-29.

### 3.4. q-PCR Detection of Streptococcus sp VT_162 in Two Validation Cohorts

**Figure 5:**
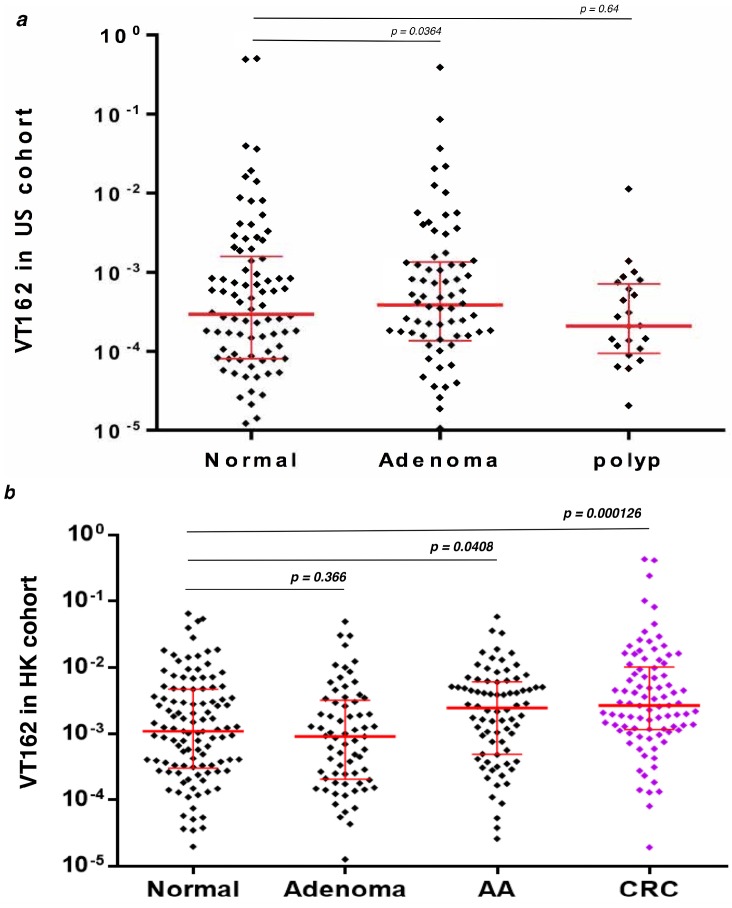
*Streptococcus* sp. VT_162 16S ribosomal RNA gene quantitative amplification (q-PCR) data in two validation cohorts. The Mann-Whitney test was used for *p* value calculation: (**a**) US cohort: 90 normal, 24 polyps (Hyperplastic), and 74 adenomas from African American patients, (**b**) HK cohort: 85 normal, 69 adenoma, 78 advanced adenoma (AA) and 88 cancers (CRC) from Chinese patients in Hong Kong.

*Streptococcus* sp VT_162 16S rDNA analysis in African American and Hong Kong stool samples validation sets revealed no statistical significance between normal vs. Polyps and normal vs. Adenomas in both sets of samples. However, the Mann-Whitney analysis revealed a statistically significant difference between normal vs. advanced adenoma (*p* = 0.041) and normal vs. Cancer (*p* = 0.00013) in the Hong Kong stool samples (Figure 5). No advanced adenoma or cancer patients’ stool samples were available for African Americans.

## 4. Discussion

Several studies have recently addressed the issue of microbiota participation and potential roles in colon oncogenic transformation [35,36,37,38,39]. Our present study extends those findings with the specific goal of finding gut microbiota markers with diagnostic value, taking into consideration the likely participation of several bacterial actors at once in a process as complex as cancer in the colon that harbors the most diverse microbiome in the human body. We report here the presence of distinct stool metabolomic profiles in patients with colon adenomas when compared to those from healthy subjects. Partial Least Squares Discriminant Analysis (PLS-DA) revealed a close clustering of the adenoma samples’ metabolomes, further confirming different metabolite exposure in the colon mucosa of patients prone to develop colonic lesions. Short-chain fatty acids (SCFAs) were found to be more prevalent in patients with a low risk of colon cancer; other differences were also noted and were assigned to the role the microbiota plays in food processing [52]. In our samples, butyrate, acetate and propionate were more prevalent in the adenoma samples; however, many amino acids (lysine, glycine, valine and threonine) along with glucose, fatty acids and glycerol were higher in normal samples. While the SCFAs distinct presence in the adenoma samples goes against general wisdom, the low levels of amino acids, glucose and glycerol might play a role in colon homeostasis disruption as this creates an environment with fewer available nutrients to the colonocytes. Dai et al. reported that select amino acids are rapidly utilized by single bacteria such as *Esherichia coli*, *Klebsiella* sp. and *Streptococcus* sp. and bacterial mixtures, in a bacterial species and gut segment-specific manner [53]. As such, the amino acid abundance in the analyzed samples is likely reflective of different microbiota’s composition. Based on our results, it seems that the adenoma patients’ microbiota is more efficient at using amino acids than normal patients’ microbiota, which leads to a colonic environment that is depleted of essential amino acids in adenoma patients. Our finding highlights the potential use of amino acid quantification as a tool for detecting colon cancer presence or predisposition. Indeed, Yatabe et al. [54] previously reported the development of an Amino acids Index Cancer Screening (AICS) test that allowed the early detection of colon cancer in patients without clinical symptoms. Amino acids among other nutraceuticals have already been used as supplements to modulate the gut microbiota with the goal of reducing inflammation and maintaining colon homeostasis [55]. This might correspond to a first line of intervention that might reduce colon neoplastic incidence in this population.

The analysis of the colon cancer samples revealed that at the phylum level, *Firmicutes* and *Fusobacteria* were more prevalent in cancer tissues, while *Bacteroides*, *Proteobacteria*, and *Verrucomicrobia* were prevalent in the matched normal tissues (Figure 2). At the genus level, *Streptococcus*, *Prevotella*, *Fusobacteria*, *Lactobacillus*, *Veillonella*, *Gemella*, *Enterococcus* and *Actinomyces* were more strongly represented in the cancer samples when compared to their matched normal samples (Figure 2b,c). It is most striking that most differences between cancers and matched normal samples were primarily noted for *Fusobacteria*, *Prevotella* and *Streptococcus* bacteria. At the individual level, *Fusobacteria* 16S rRNA gene sequences were more prevalent in the cancer vs. matched normal in 7 out of 10 patients. The remaining three had almost the same amount. *Fusobacteria* were reported as prevalent in colon cancer tumors when compared to normal matched tissues [56]. Since then, several studies attempted to determine whether *Fusobacteria* drive the oncogenic process or merely benefit from it. Our findings presented here with stool samples 16S rRNA gene data do not substantiate an early role of *Fusobacteria* as they were barely detectable in the adenoma stool samples. Also, our previous work on polyp patients [40] did not reveal any *Fusobacteria* presence in colon polyps’ stool samples. One might assume that this bacterium is primarily an adherent bacterium that will be primarily detected in colon biopsies rather than in stool samples. Indeed, McCoy et al. reported an association of *Fusobacteria* with colon adenoma tissues [57]. However, a recent study that analyzed *Fusobacteria* presence at different stages of the oncogenic path found that the bacterium becomes more significant in high-grade dysplasia stages, not before [58]. Moreover, the levels of *Fusobacteria* in the stools did not correlate with their levels in the cancer or advanced adenoma tissues of the same individuals [58], making this bacterium an unlikely good marker to be used for stool-based non-invasive CRC screening. However, it was shown that patients with high levels of *Fusobacteria* in their colon have lower survival, making it a potentially good prognostic marker [58].

The second major group of bacteria that showed major differences between cancers and matched normals was *Streptococcus.* These strains were more prevalent in the cancer samples vs. matched normals in all pairs of samples as well as when combined. This was true for all detected *Streptococcus* OTUs. In the adenoma stool samples, the two major detected *Streptococcus* bacteria, namely *Streptococcus mitis* et rel. and *Streptococcus bovis* et rel., showed higher prevalence in the adenoma samples when compared to the healthy subjects’ stool samples’ microbiota. This finding defines a directional change that seems to be consistent in the two sets of analyzed samples. In our previous study on colon polyp patients’ stool samples [40], *Streptococcus bovis* was detected; however, the difference between the polyp vs. normal was not as pronounced as the one observed between adenomas vs. healthy subjects or cancers vs. matched normals, reported here. As such, the *Streptococcus* gradual prevalence in both adenoma stool samples and colon cancer tissues seems to be a firm finding from the above results. Indeed, several reports have cited an association of *Streptococcus* strains with colon cancer occurrence [59,60,61,62]. *Streptococcus* bacterial strains are known as a large and dynamic component of the small intestine [63]. Would this resurgence in colon cancer tissues and adenoma stool samples correspond to a bacterial translocation with deleterious effects on colon homeostasis? This remains to be further explored. It will also be interesting to see if *Streptococcus* strain resurgence in the colon is involved in the amino acids shortage reported in the metabolomic analysis. Indeed, *Streptococcus* strains, among other bacteria, have been described as rapid utilizers of amino acids [53,64].

While our intent is to develop a stool-based screening test for CRC, a recent publication reported the prevalence of *Fusobacteria*, *Prevotella* and *Streptococcus* bacteria in laryngeal carcinomas from throat microbiota analysis [65]. These same bacteria were prevalent in our cancer specimens. Their association with laryngeal cancer and colon cancer might open the door for an upper GI flora analysis—e.g., oral flora—for the assessment of cancer risk over the GI tract, rather than relying on stool samples for such a goal.

Since our goal for this study was to define a panel of bacterial markers for non-invasive colorectal cancer screening tests and because of bacterial genomic plasticity, the 16S rRNA gene description is not always sufficient to achieve such goals. The mapping of metagenomic stool sequencing reads against the tissue samples identified bacterial genes led to 30 tissues’ microbiota genes, 6 of which were shown to have a discriminative power between adenoma vs. healthy subjects on one hand and cancer vs. matched normals on the other hand. Similarly, mapping the reads from the tissue samples to annotated sequences from stool sample data led to the identification of 4233 genes. Only 8 of these genes displayed a discriminative power between adenoma vs. healthy subjects and between cancers vs. matched normals. The 14 combined discriminatory sequences (6 from tissues and 8 from stool metagenomic data) led to 9 unique sequences after the removal of 5 duplicates. These sequences were proven to have a statistically significant potential in adenoma vs. healthy subjects’ stool samples and a striking, although not significant one, in cancer vs. matched normal samples. This finding is of utmost relevance to the goal that we set for our study since this will set the foundation for stool-based non-invasive screening test development at the preneoplastic stages.

These sequences mapped to *Streptococcus* sp. VT_162, *Acinetobacter baumanii* AC12 and *Sphingomonas* sp. PM2-P1-29. Neither *Acinetobacter* nor *Sphingomonas* showed differences between adenomas vs. healthy subjects or cancers vs. matched normals. However, as stated above, the *Streptococcus* differences at the 16S rRNA gene level were noted in both sets of data and were associated with the diseased samples (cancers and adenomas). Our findings were further validated through the use of highly specific *Streptococcus* sp. VT_162 primers and a probe in q-PCR experiments from independent cohorts. 16S rDNA detection was not significant in polyps and adenomas samples but was statistically significant in advanced adenoma (*p* = 0.041) and cancer (*p* < 0.00013) samples when compared to normal stool samples. These findings are of major significance knowing that these significant *p* values were obtained with samples from the Hong Kong validation cohort while the bacterium was identified in African American patients.

*Streptococcus* sp. vt_162 is a bacterium that was first isolated from the saliva of pediatric oncohematology patients [66]. The fact that this bacterium has been isolated in such a context gives further credibility to our findings. While the rapid amino acid using *Streptococcus* strains were consistently present in the adenoma and cancer samples at the 16S rRNA gene and metagenomics levels, the metagenomic functions that associate with amino acids metabolism in the analyzed samples showed no significant differences in adenoma samples compared with healthy subjects.

It is noteworthy that *Fusobacteria* that have been found in our study and many others as associating with colon cancer are agents of periodontal disease [67]. The fact that both *Streptococcus* sp. VT_162 and *Fusobacteria* are oral bacteria already known for their involvement in hemoncology and periodontal inflammation might be strong evidence for a possible involvement in colon oncogenic transformation. As reported above, these two groups of bacteria along with *Prevotella* have already been associated with laryngeal cancer [65], and as such, this group might potentially be used for oral microbiota assessment of CRC risk.

These findings will need to be validated in a larger population of patients that include different stages of the carcinogenic process and different ethnic backgrounds to establish the specificity and sensitivity of the discovered markers. This study also stresses the possible use of oral flora as a potential surrogate for assessing colorectal and gastrointestinal cancers’ risk among other associated health disorders.

## Supplementary Materials

The following are available online at www.mdpi.com/2073-4425/8/11/314/s1.
