# Supplementary material for: A Microbiomic Analysis in African Americans with Colonic Lesions Reveals Streptococcus sp.VT162 as a Marker of Neoplastic Transformation
Source: Genes (Basel). 2017 Nov 9;8(11):314. doi: 10.3390/genes8110314 (PMC5704227; doi:10.3390/genes8110314)
Supplement: Supplementary file 1 [file genes-08-00314-s001.pdf]

SUPPLEMENATRY TABLES:

Suppl. Table 1: Bins and library matched metabolites that distinguished adenoma patients from healthy subjects. The bin value is the center of the bin region. Directionality is based on the loadings plot in the PLS-DA (ncreased means that the bin/ metabolite is increased (highly represented) in the adenoma group when compared to the healthy controls.

Bin	Metabolite	VIP	Direction	Bin	Metabolite	VIP	Direction
0.62	Unknown	1.4	Increased	2.22	Cholate	1.0	Increased
0.86	Caprate, Valerate	1.5	Increased	2.34	Glutamate	1.0	Increased
0.90	Isovalerate	2.1	Increased	2.54	Unknown	1.6	Increased
0.94	Leucine, Isoleucine	1.8	Decreased	2.90	Unknown	1.6	Increased
0.98	Valine	1.3	Decreased	3.02	Lysine	1.2	Decreased
1.02	Valine	1.4	Decreased	3.10	Phenylalanine, Unknown	1.5	Increased
1.06	Propionate	1.5	Increased	3.14	Unknown	1.6	Increased
1.14	Lipids, fatty acids	1.7	Decreased	3.22	Glucose, O-Phosphocholine	3.5	Increased
1.18	Isopropanol, fatty acids	1.5	Decreased	3.26	Phenylalanine, Unknown	2.3	Decreased
1.30	Lactate, Threonine	1.0	Increased	3.54	Glycine, Glycerol	1.3	Decreased
1.46	Alanine, Unknown	1.6	Increased	3.58	Valine, Threonine	1.2	Decreased
1.54	Butyrate	1.8	Increased	3.62	Unknown	7.7	Decreased
1.70	Leucine	1.1	Increased	3.66	Unknown, Isoleucine	5.8	Decreased
1.74	Leucine	1.4	Increased	3.70	Unknown	3.7	Decreased
1.78	Unknown	1.0	Increased	3.74	Lysine, Isoleucine	1.2	Decreased
1.90	Acetate	3.4	Increased	3.78	Glycerol	1.3	Decreased
2.14	Butyrate	1.9	Increased	3.82	Glucose	1.3	Decreased
2.18	Propionate	1.5	Increased	4.02	Fatty acids	1.1	Decreased

Suuppl. Table 2: Summary of metagenomic data for all analyzed samples: a) Cancers and matched Normals, b) Adenomas and Healthy subjects' stool samples.

a)

Sample#CC	Location	Age	gender	Age categories	MG-RAST ID	Total read base-pairs	Total number of reads	Number of reads after de-replication	Number of reads not mapped to human genome	Number of de-novo assembled genes	Number of reads mapped to the Combined Gene Catalog
1024N	Right	73	F	>60	4537962	1,644,581,250	10,666,848	7,152,563	3,257,902	26,554	189,655
1024T	Right	73	F	>60	4537963	533,190,597	2,343,778	1,972,888	1,389,222	13,613	2,482
1028N	Left	42	M	<=50	4537964	1,753,759,640	10,579,432	7,994,209	4,985,633	31,235	159,828
1028T	Left	42	M	<=50	4537965	1,827,358,119	11,166,023	8,504,150	4,912,783	28,176	76,387
1029N	Right	51	F	<=60	4537966	2,212,344,601	13,596,567	11,091,440	6,716,519	30,661	137,705
1029T	Right	51	F	<=60	4537967	1,842,141,313	11,096,026	7,331,429	4,474,683	64,339	104,300
1038N	Left	54	M	<=60	4537968	1,878,748,442	11,413,547	9,123,450	5,323,645	26,838	62,772
1038T	Left	54	M	<=60	4537969	1,867,594,574	11,329,181	8,990,793	5,528,961	35,263	79,057
1053N	Right	50	F	<=50	4537970	2,016,157,252	12,756,861	9,929,123	6,028,666	14,410	97,227
1053T	Right	50	F	<=50	4537971	1,831,866,894	11,318,160	9,561,945	5,500,359	16,638	55,088
1054N	Right	53	M	<=60	4537972	2,203,954,960	13,565,337	13,165,786	1,956,110	391	26,906
1054T	Right	53	M	<=60	4537973	2,188,956,703	13,206,838	9,283,259	5,500,271	61,272	107,598
1057N	Left	88	M	>60	4537974	1,793,513,844	10,896,395	8,653,023	4,888,216	22,891	121,339
1057T	Left	88	M	>60	4537975	2,318,458,337	15,845,692	10,974,275	6,720,687	26,004	91,866
1059N	Right	60	F	<=60	4537976	2,025,344,413	12,358,271	9,833,739	5,320,384	33,359	78,236
1059T	Right	60	F	<=60	4537977	1,994,857,882	12,004,025	9,244,806	5,562,342	41,042	78,848
1060N	Left	53	F	<=60	4537978	2,256,852,278	13,603,661	10,288,812	6,197,098	60,460	202,474
1060T	Left	53	F	<=60	4537979	1,976,386,826	12,238,973	9,951,700	5,115,576	21,693	65,643
1065N	Left	41	M	<=50	4537980	2,025,558,322	12,369,232	11,901,313	1,686,804	2,063	25,249
1065T	Left	41	M	<=50	4537981	2,652,460,630	15,965,647	11,367,671	7,113,107	82,025	122,378

b)

sample CC#	Age	Gender	Location categories	Diagnosis	Total read base-pairs	Total number of reads	Number of reads after de-replication	Number of reads not mapped to human genome	Number of de-novo assembled genes	Number of reads mapped to the Combined Gene Catalog
CC0839	64	M	Right	Adenoma	727,169,344	4,414,873	4,047,242	4,046,313	52,828	1,401,974
CC0872	81	F	Right	Adenoma	294,228,737	1,806,268	1,586,477	1,586,103	10,755	546,277
CC0883	71	M	Left	Adenoma	315,809,466	1,941,435	1,755,506	1,754,900	22,618	584,649
CC0885	57	F	Right	Adenoma	278,928,798	1,808,625	1,616,635	1,615,619	17,491	329,503
CC0899	56	M	Right	Adenoma	14,751,107	229,185	119,562	119,337	8	55,206
CC0901	53	F	Left	Adenoma	635,650,860	4,107,892	2,690,406	2,688,671	8,073	1,069,009
CC0911	53	F	Left	Adenoma	587,523,210	3,621,581	3,334,484	3,333,562	40,755	1,539,671
CC0944	53	M	Right	Adenoma	627,105,499	3,868,823	3,347,785	3,347,097	40,548	1,786,970
CC0995	59	F	Right	Adenoma	445,737,297	2,741,790	2,508,848	2,508,654	29,133	1,143,429
CC0904	82	F	Left	Adenoma	550,743,973	3,457,852	2,963,171	2,961,799	30,207	1,041,125
CC0868	54	F	none	Normal	396,371,168	2,439,250	2,199,010	2,196,432	34,296	1,116,424
CC0890	71	F	none	Normal	313,375,574	1,921,863	1,789,807	1,789,775	12,241	827,443
CC0895	54	F	none	Normal	482,477,547	2,944,792	2,385,845	2,385,670	29,001	858,679
CC0908	64	F	none	Normal	283,650,684	1,704,845	1,455,741	1,454,499	19,109	463,351
CC0914	59	M	none	Normal	452,350,481	2,749,271	2,498,120	2,495,397	30,905	1,239,917
CC0929	59	F	none	Normal	376,566,289	2,309,525	2,121,427	2,120,809	25,829	494,802
CC0961	59	F	none	Normal	374,300,885	2,298,641	2,045,454	2,044,955	19,397	812,180
CC0981	54	F	none	Normal	314,161,995	1,946,234	1,713,667	1,695,567	12,410	698,023
CC0990	58	M	none	Normal	693,253,294	4,227,227	3,597,911	3,597,782	34,019	1,256,180
CC1068	73	M	none	Normal	443,270,953	2,708,846	2,566,677	2,566,466	25,265	1,277,085
